# Twenty-five-year atraumatic restorative treatment (ART) approach: a comprehensive overview

**DOI:** 10.1007/s00784-012-0783-4

**Published:** 2012-07-24

**Authors:** Jo E. Frencken, Soraya Coelho Leal, Maria Fidela Navarro

**Affiliations:** 1 Department of Global Oral Health, College of Dental Sciences, Radboud University Nijmegen Medical Centre, Philips van Leijdenlaan 25, 6525 AX, Nijmegen, P.O. Box 9101, 6500 HB Nijmegen, The Netherlands; 2 Department of Pediatric Dentistry, School of Health Sciences, University of Brasília, Brasilia, Brazil; 3 Department of Dental Materials, Endodontics and Restorative Dentistry, Bauru Dental School, University of São Paulo, Bauru, Brazil

**Keywords:** Atraumatic restorative treatment, Glass ionomer, Restoration, Fissure sealants, Review article, Dental public health

## Abstract

**Background:**

The atraumatic restorative treatment (ART) approach was born 25 years ago in Tanzania. It has evolved into an essential caries management concept for improving quality and access to oral care globally.

**Results:**

Meta-analyses and systematic reviews have indicated that the high effectiveness of ART sealants using high-viscosity glass ionomers in carious lesion development prevention is not different from that of resin fissure sealants. ART using high-viscosity glass ionomer can safely be used to restore single-surface cavities both in primary and in permanent posterior teeth, but its quality in restoring multiple surfaces in primary posterior teeth cavities needs to be improved. Insufficient information is available regarding the quality of ART restorations in multiple surfaces in permanent anterior and posterior teeth. There appears to be no difference in the survival of single-surface high-viscosity glass-ionomer ART restorations and amalgam restorations.

**Discussion:**

The use of ART results in smaller cavities and in high acceptance of preventive and restorative care by children. Because local anaesthesia is seldom needed and only hand instruments are used, ART is considered to be a promising approach for treating children suffering from early childhood caries. ART has been implemented in the public oral health services of a number of countries, and clearly, proper implementation requires the availability of sufficient stocks of good high-viscosity glass ionomers and sets of ART instruments right from the start. Textbooks including chapters on ART are available, and the concept is being included in graduate courses at dental schools in a number of countries. Recent development and testing of e-learning modules for distance learning has increasingly facilitated the distribution of ART information amongst professionals, thus enabling more people to benefit from ART. However, this development and further research require adequate funding, which is not always easily obtainable. The next major challenge is the continuation of care to the frail elderly, in which ART may play a part.

**Conclusion:**

ART, as part of the Basic Package of Oral Care, is an important cornerstone for the development of global oral health and alleviating inequality in oral care.

## History

The request for a comprehensive overview on the atraumatic restorative treatment (ART) is very timely. It is 25 years since the results of the first pilot study, in which excavators were used to remove soft, completely demineralised dentine from painful dentine cavities that were then filled with polycarboxylate cement, were presented at an international meeting in Dar es Salaam, Tanzania. Of the 28 treated teeth, only one needed to be extracted. All the others, although showing visible surface wear of the cement, functioned well without pain or negative symptoms, after 9 months. The patients were very pleased that extraction of the teeth, which was the expected and common treatment for painful teeth in the country, had not happened. Thereafter, the treatment procedure evolved through the 1980s and 1990s, into the twenty-first century. The restorative material changed to glass ionomer. Not only was glass ionomer used to restore cavities in stress-bearing situations, not common in those days, but it was also used to seal caries-prone pits and fissures. Even the use of hand instruments did not remain restricted to cleaning relatively large cavities, as was the case in the Tanzanian pilot study. The technique was refined. The orifice of small dentine cavities could be enlarged with a hatchet and/or an enamel access cutter. This operational advancement expanded the application of the unconventional preventive and restorative care concept that became known in the early 1990s as the ART approach.

In the early years, a medium-viscosity glass ionomer [1, 2] was the only suitable type available. The insertion of this type of glass ionomers in stress-bearing tooth surfaces triggered manufacturers to develop a more wear-resistant glass ionomer. These so-called high-viscosity glass ionomers were introduced in the mid-1990s and are still the type of glass ionomer required with ART.

The early propagators of ART realised its potential for global oral health (personal communication). As is still the case today, the large majority of cavitated dentine lesions went untreated at the time when ART was born. This situation not only prevailed in low- and middle-income countries but was also present in high-income countries. There are a number of factors that can explain this unwanted situation in each of the world's countries. However, the unconditional introduction of the traditional treatment scheme developed for use in Western countries, into low- and middle-income countries, is considered a major reason for the low level of preventive and restorative care provided in many communities in these countries over the last decades [3]. To improve this situation, the traditional treatment scheme needed to be drastically changed and ART could make that difference. In order to maximise the potential benefits, aspects of ART needed to be researched from the very beginning. Consequently, a research agenda were developed and updated at various international dental congresses [4–6]. This initiative had led to a total of 260 publications on various aspects of ART in PubMed by early May 2012, to a number of textbooks on ART [7, 8] and to chapters on ART in textbooks on minimal intervention dentistry [9], preventive and community dentistry [10], cariology [11] and paediatric dentistry [12].

## The ART concept

Currently, ART is defined as a minimally invasive care approach in preventing dental caries and stopping its further progression. It consists of two components: sealing caries-prone pits and fissures and restoring cavitated dentine lesions with sealant-restorations [7]. The placement of an ART sealant involves the application of a high-viscosity glass ionomer that is pushed into the pits and fissures under finger pressure. An ART restoration involves the creation of sufficient access to the cavity for the removal of soft, completely demineralised (decomposed) carious tooth tissues with hand instruments. This is followed by restoration of the cavity with an adhesive dental material which simultaneously seals any remaining pits and fissures that remain at risk. Opening the cavity with rotating instruments, followed by cleaning it with hand instruments and restoring it with an adhesive restorative material, is not considered ART nor can calling it modified ART be justified [13]. Meanwhile, the use of ART is no longer restricted to low- and middle-income countries. There is evidence that ART application has spread to very many countries in the world. In many countries, ART is part of the dental curriculum [14, 15]. Many private practitioners in BRIC and Western countries use it to complement other treatment concepts in the provision of dental care to their clientele (Brazil, Japan, Netherlands, UK, USA). Because it seems that the use of ART is growing in many parts of the world, this was considered an appropriate time for presenting and discussing the knowledge, experiences and study results regarding various aspects of ART. That is the aim of the present publication.

## The “atraumatic” component of the ART approach

Since its start, the originators of the ART approach noticed that the technique had a potential to cause less discomfort to the patient and to be less invasive to the dental tissues than the conventional approach. The patient's acceptance of ART was verified by Mickenautsch and Rudolph [16], who observed that both children and adults receiving ART restorations responded very positively to the treatment. They ascribed this reaction to ART's “patient friendly” properties. Dentists also seemed to approve the “new” approach. Among the main reasons given were those related to the patient's comfort: the reduced use of local anaesthetic and absence of the noisy drill and suction [17]. Dental students' perceptions, after receiving a special training on ART and applying it to their patients, were in agreement with those of the dentists. The fifth-year students reported perceptions related to dental anxiety, minimal loss of tooth tissues and to the fact that they could clearly see patients' expressions changing from fearful to more relaxed as the most relevant aspects of the ART approach. These experiences contributed to an increase in their confidence as operators [18].

However, analysis of clinical trials that have compared patients' comfort during ART and the conventional approach using different restorative materials revealed inconclusive results. Basically, these studies investigated two aspects related to the patients' comfort: dental pain and dental anxiety. A summary of these studies' outcomes is presented in Table 1. All studies, except the one from Mickenautsch et al. [24] that also included adults, were performed on children. From the seven studies retrieved, three showed no difference between the two types of treatment in levels of anxiety or pain, while the other four suggested that ART was found to be less painful and caused less dental anxiety. What could be the explanation for these controversial results? Besides methodological aspects, apparently, the outcomes were also influenced by the operators' level of specialisation and/or skills in handling anxious children. The studies from Topaloglu et al. [23] and de Menezes Abreu et al. [21, 22], in which no difference in levels of dental anxiety and dental pain were observed, were performed by paediatric dentists. In the studies that favoured ART [19–21, 23], all operators, but the one from de Menezes Abreu et al. [21], were non-paediatric dentists (general practitioners, dental therapists or dental students). However, the latter study had included children younger than 6 years, and all those given the conventional treatment received local anaesthesia and the restorations were performed under rubber dam isolation. It is not unrealistic to argue that age and the use of the needle and that of rubber dam might have influenced children's perception of pain.

**Table 1:** Overview of studies having assessed dental anxiety and dental pain between the ART and the traditional treatment approach

Reference	Comparison	Age	Operator background	Variable measured	Conclusion
[19]	ART vs rotary instruments	6-year-old children	Dental students and dentists	Discomfort:	ART caused less discomfort
				-Heart rate and modified Venham index (observations)	
[20]	ART vs rotary instruments	6–16 years old	Dentists	Pain:	ART caused less pain
				-Questions: did you feel any pain during treatment?	
[21]	ART vs rotary instruments	4–7 years old	Pedodontist specialist	Pain:	ART caused less pain
				-Wong–Baker FACES Pain Rating Scale	
[22]	ART vs rotary instruments vs ultraconservative treatment	6–7 years old	Pedodontist specialist	Pain:	-No difference in levels of pain among treatments
				-Wong–Baker FACES Pain Rating Scale	-Local anaesthesia was more frequent given in the rotary instrument group
[23]	-ART vs rotary instruments	6–7 years old	Pedodontist specialist	Anxiety:	No difference in levels of anxiety between treatments
	-ART vs ART with Carisolv			-Venham Picture Test	
					
[24]	ART vs rotary instruments	Children and adults	Dentists and dental therapists	Anxiety:	Both children and adults treated with the ART were less dental-anxious
				-Children's fear survey schedule	
				-Corah's dental anxiety scale	
[25]	ART vs rotary instruments vs ultraconservative treatment	6–7 years old	Pedodontist specialist	Anxiety:	No difference in levels of anxiety among treatments
				-Facial Image Scale	

In light of all these aspects, it can be hypothesised that the behaviour management provided by a paediatric dentist may overcome much of the discomfort that a child can feel independently of the restorative treatment approach. On the other hand, it can be argued that ART could be a facilitator for good behaviour management when the dentist is not so skillful in dealing with children. For those very young, ART is the best treatment option, whether the operator is a specialist or not [21], as age is associated with dental behaviour management problems; the younger the child the more behaviour problems are expected [26]. With regard to adult patients, there is only one study in which the impact of ART on patients' comfort was tested [24]. It indicates that ART caused less anxiety than the traditional approach using rotary instruments.

Another atraumatic aspect of the ART approach that should be also taken into account refers to its potential to be less invasive to the dental tissues. Following the concept of minimal intervention dentistry (MID), only decomposed dentine needs to be removed in order to stop carious lesion progression. This then leads to the question as to which method removes decomposed dentine best. In vitro studies have shown that, among the common caries excavation methods tested, hand excavation was the best method, in terms of combined efficiency and effectiveness, for cleaning of occlusal cavities in primary [27] and permanent teeth [28]. It is obvious that hand instruments, unlike rotary instruments, have a limited ability to remove sound tooth tissues. It is therefore no surprise that single-surface cavities prepared by hand instruments as part of the ART approach were significantly smaller than those prepared through rotary instrumentation [29].

The ART potential as an atraumatic management approach for cavitated dentine carious lesions for both children and adults has already been discussed [30]. However, well-designed trials are still needed to confirm this conclusion as well as testing the influence of the type of operator on patients' behaviour.

## Survival of ART sealants and ART glass-ionomer restorations

Two published meta-analyses have reported on the survival of ART sealants and ART restorations [31, 32]. The former meta-analysis concluded that medium-viscosity glass ionomers should not be used with ART anymore. The latter meta-analysis, including data until February 2010, showed cumulative survival rates for single-surface and multiple-surface ART restorations in primary teeth over the first two years as being 93 and 62 %, respectively. Cumulative survival rates for single-surface ART restorations in permanent teeth over the first three and five years were 85 and 80 %, respectively. Only three studies were available that had reported on multiple-surface ART restorations in permanent teeth resulting in a 1-year survival rate of 86 %.

During the period February 2010 to November 2011, seven additional studies reporting on ART restorations have been published. Using the inclusion criteria of the meta-analysis [32], results of four studies could be used to update the survival rates of ART restorations. Regarding single-surface ART restorations in posterior primary dentitions, one study [33] was in line with the outcome of the meta-analysis (95 %) whereas the other study showed a somewhat lower result (80 %) after 1 year [34]. The study by Deepa and Shobha [33] reported an 89 % survival of 1-year-old multiple-surface ART restorations in the primary dentition, compared to the 71 % survival rate after 1 year resulting from the meta-analysis. Regarding single-surface ART restorations in the posterior permanent dentitions, one of the added studies showed a 2-year survival of 94 % [35], which is in line with the result from the latest meta-analysis on ART (93 %). The fourth included study is the longest run reported ART study [36]. Data were assessed using a slightly modified version of the ART restoration criteria [11] and the original United States Public Health Service (USPHS) criteria [37]. After 10 years, using the ART restoration criteria, 65 % of single-surface ART restorations in posterior permanent teeth had survived, so did 31 % of multiple-surface ART restorations in this group of adults. The survival results, using the USPHS criteria, were completely different. After 10 years, 87 and 58 % of single-surface and multiple-surface ART restorations had survived, respectively. This big difference is basically due to two aspects: (1) the fact that the original USPHS criteria fail restorations only when the dentine is visible and (2) the original USPHS criteria do not fail a tooth re-restored by a non-study team dentist. The ART criteria fail a restoration when 0.5 mm of enamel is visible at the margin of the restoration and fail a re-restored tooth placed by an outside dentist. From the above, it emerges that had the USPHS criteria been applied to assess ART restorations instead of the ART restoration criteria, the survival rates of ART restorations would have been higher than reported. For the Zanata et al. [36] study, the difference was 22 and 27 % for single-surface and multiple-surface ART restorations after 10 years, respectively.

We can conclude the following:

- ART sealants have a high caries preventive effect;
- ART using a high-viscosity glass ionomer can safely be used in single-surface cavities in both primary and permanent posterior teeth;
- ART using a high-viscosity glass ionomer cannot be routinely used in multiple-surface cavities in primary posterior teeth;
- Insufficient information is available for conclusions about ART restorations in multiple surfaces in permanent posterior teeth and in anterior teeth in both dentitions; and
- The ART restoration criteria, used in most ART studies, are more stringent than the original USPHS criteria and lead to lower survival result reports than would be obtained when the USPHS criteria are used.

Overall, the ART approach can routinely be used in single-surface cavities in both primary and permanent posterior teeth.

## ART restorations vs traditional restorations

### Primary dentition

The number of studies that have compared ART with amalgam restorations in the primary dentition is low. The systematic review of Mickenautsch et al. [38] concluded that there was no difference between the two types of restoration.

ART high-viscosity glass-ionomer restorations have also been compared to conventional composite resin restorations in primary molars. Using a split-mouth study design, Ersin et al. [39] found no statistically significant difference between class I ART (96.7 %) and comparable composite resin (91 %) restorations and between class II ART (76.1 %) and comparable composite resin (82 %) restorations after 2 years. However, as was stated, the number of studies is too low to draw any conclusions especially for multiple-surface cavities.

### Permanent dentition

Single-surface ART restorations placed in the permanent dentition have been compared to comparable amalgam ones, but not to composite resin restorations. In a meta-analysis based on five studies, Frencken et al. [40] concluded that there appeared to be no difference in survival rates between single-surface ART restorations using glass-ionomer and amalgam restorations in permanent teeth over the first three years. Using seven studies, three related to primary and four to permanent teeth, in a systematic review, Mickenautsch et al. [38] concluded that the longevity of all types of ART restoration is equal to or greater than that of equivalent amalgam restorations for up to 6.3 years and is site dependent. This systematic review shows that, with respect to survival rates, the ART approach using high-viscosity glass ionomers produces restorations comparable to those of the conventional approach using amalgam in posterior permanent teeth. This conclusion is different from that presented in the recently launched WHO report about dental materials [41], in which glass-ionomer restorations were given a much lower survival rate than amalgam restorations. Notwithstanding the outcome of the systematic review [38], more information is necessary before the ART approach can be recommended for use in multiple-surface and anterior cavities.

## ART and secondary caries

One of the concerns expressed in the early years of ART development was the expected high percentage of restoration failures due to the development of so-called secondary caries because decomposed dentine is sometimes left behind in the cavity after ART manual cleaning. However, dentine carious lesion development at the margin of ART glass-ionomer restorations was low [36, 42–45]. This finding is supported by the results of the systematic review which showed that glass ionomer had a higher caries-preventive effect than amalgam for restorations in permanent teeth, with no difference in primary teeth [46]. These results had not been expected when the ART approach was conceived 25 years ago. However, they show that the ART approach seems to be a realistically practical option for managing dental caries, whether applied in the dental surgery or in the field.

## Sealants

Three structured reviews on the caries-preventive effect of sealants in pits and fissures did not analyse differences between glass-ionomer and resin-based sealants [47–49]. One systematic review concluded that there is no evidence that either resin-based or low- and medium-viscosity glass-ionomer sealant material is superior in preventing dentine lesion development in pits and fissures over time [50]. This finding was supported by another systematic review [51].

It is fair to conclude that low- and medium-viscosity glass-ionomer and resin pits and fissure sealants are equally good in preventing carious lesion development in pits and fissures.

## ART sealants

It is generally accepted that composite resin sealants are retained longer than low-viscosity glass-ionomer sealants [52, 53]. However, the low retention rates for low/medium glass-ionomer sealant materials, found in the 1980s and 1990s, are not so apparent anymore when high-viscosity glass ionomer is used to seal caries-prone pits and fissures with ART. The ART meta-analysis showed a 2- and 3-year fully and partially retention rate of ART high-viscosity glass-ionomer sealants of 82 and 72 %, respectively [32]. Only one ART high-viscosity glass-ionomer sealant study has been published since February 2010 and showed a fully and partially retention rate of 78 % after 2 years [54]. The ART meta-analysis showed a mean annual cavitated dentine lesion incidence rate, in pits and fissures previously sealed using ART, of 1 % over the first three years, which is low [32]. More studies are needed to confirm this result.

## ART and resin sealants

Only one study has been published in which ART high-viscosity sealants have been compared with light-cured resin sealants [55]. The authors concluded that the caries-preventive effect of high-viscosity glass-ionomer ART sealants was between 3.1 and 4.5 times higher than that of composite resin sealants after 3 to 5 years. What could be the reason for high caries-preventive action of glass-ionomer sealants after the material had disappeared from the pits and fissures? Studies have provided some evidence that glass-ionomer remnants might still be present in the bottom of pits and fissures that appear clinically free of sealant material [56–60]. These remnants are probably present because glass-ionomer fractures cohesively, in contrast to resin-based materials which tend to fracture adhesively [61]. The remnants may thus allow continuing exercising of their carious lesion preventive effect over a long period.

## Materials used with ART

Although most researchers have used an autocured glass ionomer to restore cavities cleaned with hand instruments, resin composite [62, 63], compomer [64] and resin-modified glass ionomer [65–67] have also been used for this purpose. Nevertheless, autocured high-viscosity glass ionomer appears to be the most appropriate adhesive restorative material for use with ART. However, many brands of glass ionomer exist and many are very cheap. They are, therefore, attractive to buy for dentists and governments with a limited budget. Personal experiences have shown that many of the cheap brands lead to poor quality sealants and restorations. A well-cleaned cavity can thus result in a poor restoration when a substandard glass ionomer is being inserted.

In vitro studies have tested various physical properties of medium- and high-viscosity glass ionomers. Almost consistently, the scores for high-viscosity glass ionomers were significantly higher than for medium-viscosity glass ionomers [68–70]. It is, therefore, correct to state that in order to obtain high survival rates of ART sealants and ART restorations, dental practitioners should use high-viscosity glass ionomers and select those that have been tested favourably in clinical studies of long duration.

## ART in young children

ART has been suggested to be the most logical and appropriate tertiary preventive measure for managing severe early childhood caries (S-ECC), not only for children in disadvantaged communities but also for those attending private practice facilities. It was thought that ART would avoid the need for major restorative dental care under either local or even general anaesthesia [71]. It is, therefore, surprising that only a few clinical studies have investigated the ART approach as a possible alternative to the conventional treatment for S-ECC.

Faccin et al. [67] investigated the survival of class I ART restorations in a group of preschool children (mean age 31 months). The treatment was provided in a dental setting by trained final year dental students using a resin-modified glass ionomer. The survival rates were high and varied from 89 to 72 %, depending on the evaluation period of 6 to 48 months, respectively. Lo and Holmgren [72] investigated ART in preschool children (mean age 5.1 years) who were treated by final year dental students in a kindergarten environment. The survival rates were high for class I (79 %) and class V restorations (71 %) and modest for class II restorations (51 %) after 30 months. The acceptance of ART was also assessed, and the authors concluded that preschool children who received ART restorations in a kindergarten environment accepted the treatment well, probably because it was provided in a nonthreatening way in a familiar setting. This outcome indicates that ART can be applied outside the traditional clinical setting, even for very young children. This is a very important finding as ART, when compared to traditional ways of managing dental caries, presents two important advantages: the low cost and its possibility for application at school premises, which may lead to an increase in dental service coverage.

In terms of ART acceptance, de Menezes Abreu et al. [21] compared ART and the traditional approach, focusing on children's reporting of pain. The study included children from 4 to 7 years old. It concluded that ART was less painful than the conventional restorative treatment (CRT) and that the youngest children complained most about pain. While all children aged 4 from the CRT group reported some level of pain, 30 % of those who received ART reported that they had felt no pain. Why? This is, most probably, because the most fear-eliciting stimuli related to the dental treatment, as pointed out by school children—the sight and sensation of an anaesthetic needle and of the drill [73]—were absent when ART was applied.

We agree with Davies [71], who affirmed that ART provides a much more acceptable introduction to dental care than “injection, drill and fill”. However, well-conducted clinical trials are required to show the benefits of the ART approach in restoring cavitated lesions in very young children, which would benefit thousands of children and families all over the world.

## ART in the elderly 

From its onset, one of the indications for the appropriate use of the ART approach concerned the elderly, particularly those living in institutions and those who are homebound [74]. Unfortunately, very few studies have investigated the potential of ART in providing dental care to these people. The first of such study was carried out amongst, on average, 75-year-old subjects who were homebound because of physical, mental or emotional problems [75]. The majority of carious lesions presented were so extensive that restorative care for these elderly people was no longer possible. After 1 year, 79 % of the ART restorations placed were considered successful. ART was well received, and the recipients were very satisfied with the care provided at home. A second study was performed on root surface carious lesions amongst, on average, 63-year-old subjects who had undergone radiotherapy. After 2 years, there was no significant difference in survival rates between ART restorations and those produced through the traditional approach, using high-viscosity glass ionomer—66.2 % vs 65.2 %, respectively [76]. The last study investigated the survival of ART restorations in root surfaces among institutionalised elderly with an average age of 78.6 years in comparison to that of traditional treatment using a resin-modified glass ionomer. The 1-year survival rate for traditional restorations was 91.7 %, and it was 87 % for ART restorations [77].

It is obvious that the potential for treating elderly patients in hospitals, institutions or their own homes, as inherent in the ART approach, has been insufficiently researched. Considering the worldwide increase of elderly people with natural dentition in the coming decades, study covering the impact of the ART approach, as part of a package of (medical) oral care for use among the elderly, should receive serious attention.

## ART in the public services

The first report that described the use of the ART approach in a public service system originated from South Africa. ART was introduced there mainly because of its appropriate economical and restorative advantages and because of its patient friendliness [16]. The adoption of ART was associated with training, research and follow-up supervision [16]. Since then, the ART approach has been proposed in several countries as an appropriate caries management concept aimed at improving the public oral health services [78] by minimizing the number of extractions, maximising the number of dental restorations and sealants [79] and increasing the coverage of addressing dental care needs in a population [15].

The Mexican experience of incorporating ART into the public service stands out as a good example [80]. It started with an ART course in 1998, followed by the development and acceptance of a National Oral Health Programme (including ART) and subsequently, in 2002, a second ART course after which the programme could commence fully. It was estimated that 2 million ART procedures were performed in the first six years of the programme, an increase of 400 % from the baseline, and that 810 dentists had been trained in ART. The restoration statistics represented an overestimation, as some 55 % of the dentists had used the drill to open cavities and then cleaned them with hand instruments. The resulting restorations cannot be considered ART restorations [13]. The Oral Health Programme was then extended to cover 100 municipalities. More ART training courses were conducted, and 1,300 dentists were trained in the use of ART. The success of the restorations in primary and permanent teeth was 82 % after 1 year. The programme is considered to have been successful because of the high number of dentists trained, the high number of restorations placed, the success rate of ART restorations and the increased administration of dental care covering populations low on the human development index [80].

According to nine chief dental officers of 10 Latin American countries, ART has been introduced into their countries' public oral health service systems [15], but the implementation is still in its infancy.

The implementation of ART in the public health services has also been researched in Tanzania. ART introduction resulted in an increase in the mean percentage of total restorations in relation to total treatment rendered, from 3.9 % at baseline to 13 % at the end of the 31-month study period [81].

The experiences in South Africa, Mexico, Tanzania, the Latin American countries and Cambodia [82] show that the proper implementation of ART in the public oral health services is mainly hampered by two factors: the availability of ART instruments and the availability of quality glass ionomers. Strategies for successful incorporation of ART into public oral health services should, therefore, include organisation of training courses in ART for trainer dentists, in addition to regular complete ART courses in countries that have already organised such courses; support for course participants through ensuring the constant supply of quality high-viscosity glass-ionomer restorative material; installation of a system for monitoring treatments provided in public oral health services; organisation of meetings for updating dental practitioners about monitored results; and promotion of cooperation between universities and the health ministries in developing the ART oral health projects [15].

Finally, introducing ART as part of the Basic Package of Oral Care [83] increases the chance for rendering essential palliative, preventive and restorative care, in addition to promotional activities, to many communities in need.

## ART and dental education

The principles of ART perfectly fit the concept of MID. The first IADR symposium on MID techniques was almost solely dedicated to the ART approach [84]. It is obvious that populations stand to benefit from ART if the approach is being taught at dental schools.

Not too many studies have reported on the incorporation of ART into the dental curriculum. A survey amongst dental schools in Brazil revealed that ART lectures were given in 95 % of responding schools [14]. ART was mainly being taught as a paediatric dentistry discipline (70 %). A full ART course was offered in 63 % of the responding dental schools. The situation in Brazil might reflect that of 10 other Latin American countries. The chief dental officer stated that ART was being taught in 74 % of dental schools in these countries and was taught most frequently as a paediatric dentistry discipline [15].

Advances in dissemination of information about ART aspects within the professional education system have been reported. An e-learning ART training model was shown to increase the knowledge of public health dentists about ART after postgraduate education, in Brazil [85]. This teaching mode might be very useful to students, dentists and educators, in under and postgraduate teaching, for standardising the way in which ART education is given [86].

The extent to which ART is being taught in dental schools in the world is not known. The fact that ART is included as a chapter in the cariology book edited by Fejerskov and Kidd [87] indicates the existence of a need for knowledge that will assist lecturers and students in studying the aspects of ART.

## Concluding remarks

During the last 25 years, the ART approach has become a major asset in global oral health. Nevertheless, a lot more ought to be done in order for oral health care to improve and become accessible to the many who do not have access or who have adequate access to oral health care. ART has risen to its present position because its originators, from time zero, emphasised the need for research into its various aspects to accompany its development. The latest research agenda regarding ART are an excellent example [88]. In conclusion, as Holmgren and Frencken [89] stated, ‘ART has served as a catalyst for a new way of thinking about oral health care. While oral health promotion through prevention remains the essential foundation of oral health, the ART approach is an important corner stone in the building of global oral health’.
